# Diagnostic dilemma in post-abortion intrauterine retention: endometrial polyps mimicking retained products of conception

**DOI:** 10.3389/fgwh.2025.1666642

**Published:** 2025-11-03

**Authors:** Wei-Fang Wu, Shi-Han Yan, Hai-Hua Xu, Chao-Bin Liu, Xi Xie, Shun-He Lin

**Affiliations:** Department of Obstetrics and Gynecology, Fujian Maternity and Child Health Hospital, College of Clinical Medicine for Obstetrics & Gynecology and Pediatrics, Fujian Medical University, Fuzhou, Fujian, China

**Keywords:** endometrial polyps, retained products of conception, incomplete abortion, hysteroscopy, misdiagnose

## Abstract

**Objective:**

This retrospective study investigated the characteristics of endometrial polyps identified during incomplete abortion management and evaluated differences between these polyps and retained products of conception.

**Methods:**

Patients with intrauterine retention within 4 months after abortion were enrolled in this study between January 2019 and December 2024. Twenty-six patients with pathologically confirmed endometrial polyps were included in the case group, while fifty-two patients with confirmed retained products of conception (RPOC) comprised the control group. The groups were matched in a 1:2 ratio based on gestational age (±1 week).

**Results:**

Twenty-six study group patients were included; 69.2% (18/26) were nulliparous. Abortions occurred in gestational age of 6–14 weeks. No polyps were identified prior to subsequent surgical intervention. Hysteroscopy was performed on 24 women. In hysteroscopic cases, no endometrial polyp was larger than 2 centimeters in size. Compared with control group, the study group had lower gravidity (1 [0–3] vs. 2 [0–8], *p* = 0.025) and lower serum β-hCG levels (3.67 [0–799.1] mIU/ml vs. 21.08 [0–901.2] mIU/ml, *p* = 0.004). Ultrasonography indicated a lower rate of abundant blood flow (7.7% vs. 46.2%, *p* = 0.001) and smaller intrauterine volume (1.93 ± 2.55 cm^3^ vs. 5.42 ± 4.94 cm^3^, *p* = 0.001) in the study group. Additionally, the study group had a significantly longer interval from pregnancy termination to subsequent surgical intervention (51.5 ± 31.7 days vs. 38.2 ± 14.9 days, *p* < 0.001).

**Conclusions:**

Endometrial polyps should be considered in stable women after abortion with intrauterine retention present with low blood flow on doppler, low β-hCG levels, and prolonged retention, especially in women with lower gravidity. Hysteroscopy is recommended for accurate diagnosis and proper management, preventing unnecessary treatment for presumed retained products of conception.

## Introduction

Endometrial polyps (EP) are benign focal overgrowths of endometrium that contain both endometrial glands and stroma (1, 2). They are common gynecologic disorder, especially in late reproductive or postmenopausal age group (3), occurring in 7.8% to 34.9% of women. Incomplete abortions are defined by the intrauterine retention of the products of conception (RPOC) after their incomplete or partial expulsion, occurring in up to 6% of pregnancies (4). Previous observational studies (5, 6) have suggested that incomplete abortions may be managed by expectant care, medical treatment or surgery. Although current evidence suggests that the possibility of endometrial polyps identified after abortion (7, 8), there is almost no data regarding distinguishing endometrial polyp from RPOC in the uterine cavity after abortion.

The present study was restricted to endometrial polyps identified after abortion. In this context, we sought to investigate whether there are any differences between endometrial polyps and RPOC. By addressing this gap, we seek to improve diagnostic accuracy and guide appropriate management.

## Materials and methods

This is a retrospective study conducted in Fujian Maternity and Child Health Hospital. The study was approved by the Ethics Committee of Fujian Maternity and Child Health Hospital (Reference No. 2023KY059).

**Figure 1:**
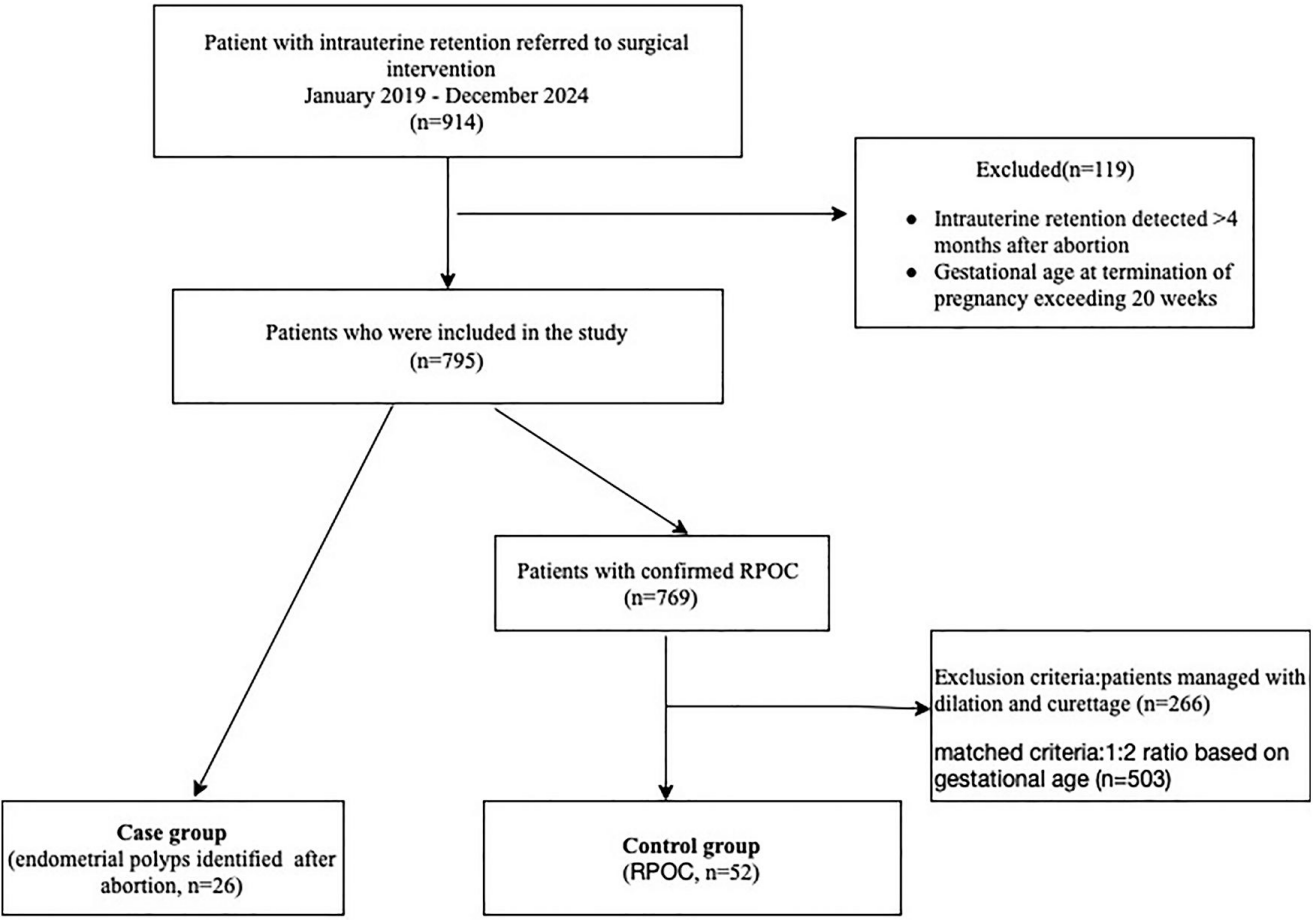
Flowchart of the study population.

The study consecutively enrolled patients who required surgical intervention for intrauterine retention identified within four months following abortion, between January 2019 and December 2024. Cases with gestational age at termination of pregnancy exceeding 20 weeks were excluded. The **case group** included patients with histologically confirmed endometrial polyps. The **control group** was selected from women with pathologically verified RPOC who underwent hysteroscopic management. To minimize confounding by hemodynamic instability, patients managed with dilation and curettage were excluded from the control group. Cases were matched to controls in a 1:2 ratio based on the gestational age at pregnancy termination, with a tolerance of ±1 week (Figure 1).

The medical records of all identified cases were extracted and evaluated. Data regarding age, gravidity, parity, presenting symptoms, serum β-hCG levels (negative threshold: <5 mIU/ml), sonographic findings, operative procedures, dimensions and localization of endometrial polyps, and pathological reports were meticulously compiled.

The removal of endometrial polyps was performed either by curettage or hysteroscopy. All hysteroscopic procedures were conducted under general anesthesia, using a rigid 5 mm hysteroscope (Storz, Germany). The distending media was normal saline and the water pressure was 100 mmH_2_O. The cervical canal and the whole uterine cavity were investigated by hysteroscopy. The location of the uterine anomalies was identified and the tissue removed with forceps. Any retained tissue in the uterine cavity was sent for pathological examination in all patients. Histopathological examination confirmed all diagnoses.

### Statistical analysis

Continuous variables were presented as mean ± standard deviation or as median (range) and were analyzed using the Student *t*-test or Mann–Whitney *U* test (intergroup comparisons), based on the results of the Kolmogorov–Smirnov test for normal distribution. Categorical data were presented as number and percentages and were analyzed using the chi-square test or the Fisher exact test, as appropriate. A *p*-value of less than 0.05 was considered statistically significant. Data processing and statistical analyses were completed using SPSS version 25.0 (IBM, Armonk, NY, USA).

## Results

During the study period, twenty-six patients were identified with endometrial polyps within four-month period subsequent to abortion. The characteristics of the study group are summarized in Table 1. The age of the patients varied between 22 and 41 years, with an average of 31.0 ± 5.5 years. The gestational age at the time of pregnancy termination ranged from 6 to 14 weeks, with an average of 8.4 ± 2.3weeks. Gravidity ranged from 0 to 3 and parity from 0 to 2. Eighteen women (18/26, 69.2%) were nulliparous. Three women conceived through assisted reproductive technology. Seven patients suffered from spontaneous abortion, 11 patients suffered from missed abortions, and 8 patients experienced induced abortions. Embryonic loss accounted for 69.2% (18/26). After abortion, 20 women experienced cessation of vaginal hemorrhage within two weeks, whereas 6 women had prolonged vaginal hemorrhage exceeding 14 days. One patient had a history of hysteroscopic polypectomy, and three patients were combined with polycystic ovary syndrome. Four patients had a history of prior missed abortion. Only three patients (3/26, 11.5%) underwent sonographic assessment prior to conception, with no sonographic evidence of endometrial polyps detected. Subsequently, sonographic evaluations in all women during gestation were uniformly unremarkable with respect to endometrial polyps.

**Table 1:** Baseline characteristics of patients with endometrial polyps identified after abortion.

Parameter	Value
Total	26
Age (year)	31.0 ± 5.5 [22–41]
Gestational age at termination of pregnancy (week)	8.4 ± 2.3 [6–14]
Abortion type	
missed abortion	11 (42.3)
spontaneous abortion	7 (26.9)
induced abortion	8 (30.8)
Gravidity	0.9 ± 1.0 [0–3]
Parity	0.5 ± 0.5 [0–2]
Nulliparous	18 (69.2)
Parous	8 (30.8)
Mode of conception	
Spontaneous	23 (88.5)
IVF-ET	3 (11.5)
Initial treatment	
Drug intervention	13 (50)
Surgical intervention	10 (38.5)
None	3 (11.5)
Vaginal hemorrhage	
≤14 days	20 (76.9)
>14 days	6 (23.1)
Serum β-hCG	
Negative	15 (57.7)
Positive	11 (42.3)
Interval between pregnancy termination and subsequent surgical intervention (day)	51.5 ± 31.7 [3–112]
Subsequent surgical intervention	
Dilatation and curettage	2 (7.7)
Hysteroscopy	24 (92.3)
Decidua shown in pathological examination	18 (69.2)

Data are presented as mean ± standard deviation [range], or number (%).

After abortion, sonographic examinations prior to surgical intervention indicated hyperechoic intrauterine lesions in 26 patients. None of endometrial polyps was identified prior to surgical intervention. The concentrations of serum β-hCG were negative in 15 (57.7%) patients, and the other 11 patients varied between 5.5 and 799.1 mIU/ml. The time interval between abortion and subsequent surgical intervention ranged from 3 to 112 days, with a median duration of 51.5 days. Hysteroscopic procedures were conducted in 24 women, while dilation and curettage interventions were executed in 2 women. None of the 24 patients subjected to hysteroscopic evacuation required additional surgical intervention. Decidua was positive in 18 cases (18/26, 69.2%). Following surgical intervention, menstrual pattern was normalized in all patient.

Table 2 presents the characteristics of 24 hysteroscopic cases in study group. The diameter of endometrial polyps ranged from 0.3 to 2.0 centimeters, with a median value of 1.05 ± 0.1 centimeters. Six cases presented with multiple endometrial polyps, while remaining cases were single endometrial polyp. The polyp was located in the upper third of the uterine cavity in 14 cases, 9 cases were located in the middle third of the uterine cavity and one case was located in the lower of the uterine cavity. Five polyps presented as pale yellow nodules, while 5 polyps presented as dark red nodules and 14 polyps presented as red nodules. Intrauterine adhesions were diagnosed in 1 patient. Decidua were positive in 16 patients. No cases of uterine perforation or hysteroscopic-related morbidity were reported.

**Table 2:** Characteristics of endometrial polyps in hysteroscopic cases.

Parameter	Value
Total	24
Size (cm, median [range]	1.05 [0.3–2.0]
≤2 cm	24 (100)
>2 cm	0 (0)
Number	
Single	18 (75)
Multiple	6 (25)
Location	
Upper third of uterine cavity	14 (58.3)
Middle third of uterine cavity	9 (37.5)
Lower third of uterine cavity	1 (4.2)
Appearance	
Pale yellow nodules	5 (20.8)
Dark red nodules	5 (20.8)
Red nodules	14 (58.3)
Intrauterine adhesions	1 (4.2)
Decidua shown in pathological examination	16 (66.7)

Data are presented as mean [range], or number (%).

Table 3 presents a comparison of the characteristics between study group and control group. There were no significant differences in age, gestational age at termination of pregnancy, abortion type, parity, mode of conception, initial treatment, vaginal hemorrhage between the two groups, prior endometrial polypectomy and polycystic ovary syndrome (*p* > 0.05). However, the study group had lower gravidity than controls (1 [0–3] vs. 2 [0–8]). The serum β-hCG levels were also significantly lower in the study group compared to the control group (3.67 [0–799.1] mIU/ml vs. 21.08 [0–901.2] mIU/ml, *p* = 0.004). Ultrasonic findings revealed a significantly lower rate of abundant blood flow in the study group (7.7%) compared to the control group (46.2%, *p* = 0.001). Additionally, the intrauterine volume was also significantly smaller in the study group (1.93 ± 2.55 cm^3^) compared to the control group (5.42 ± 4.94 cm^3^, *p* = 0.001). Furthermore, the interval between pregnancy termination and subsequent surgical intervention was significantly longer in the study group (51.5 ± 31.7 days) compared to the control group (38.2 ± 14.9 days, *p* < 0.001).

**Table 3:** Comparison of the characteristics between two groups.

Characteristic	Study group (*n* = 26)	Control group (*n* = 52)	*P* value
Age (year)	31.0 ± 5.5	31.9 ± 4.4	0.094
Gestational age at termination of pregnancy (week)	8.4 ± 2.3	8.4 ± 1.8	0.159
Abortion type			0.722
Fetal demise	18 (69.2)	38 (73.1)	
Induced abortion	8 (30.8)	14 (26.9)	
Gravidity	1 [0–3]	2 [0–8]	0.025
Parity	0 [0–2]	1 [0–6]	0.1
Mode of conception			0.685
Spontaneous	23 (88.5)	48 (92.3)	
IVF-ET	3 (11.5)	4 (7.7)	
Initial treatment			0.399
Drug intervention	13 (50)	26 (50)	
Surgical intervention	10 (38.5)	24 (46.2)	
None	3 (11.5)	2 (3.8)	
Vaginal hemorrhage			
≤14 days	20 (76.9)	40 (76.9)	1.000
>14 days	6 (23.1)	12 (23.1)	
Serum β-hCG (mIU/ml)	3.67 [0–799.1]	21.08 [0–901.2]	0.004
Ultrasound			
Abundant blood flow	2 (7.7)	24 (46.2)	0.001
Intrauterine volume (cm^3^)	1.93 ± 2.55	5.42 ± 4.94	0.001
Interval between pregnancy termination and subsequent surgical intervention (day)	51.5 ± 31.7	38.2 ± 14.9	<0.001
Prior endometrial polypectomy	1 (3.8)	1 (3.8)	1
Polycystic ovary syndrome	3 (11.5)	4 (7.7)	0.575

Data are presented as mean ± standard deviation, median [range], or number (%). Missed abortions or spontaneous abortions are grouped as fetal demise.

## Discussion

In this retrospective study, we present 26 cases of endometrial polyps identified during the management of incomplete abortion. Endometrial polyps typically present with abnormal bleeding. However, many endometrial polyps are asymptomatic and discovered incidentally for unrelated causes (9). Endometrial polyps identified in recurrent pregnancy loss are reported in some publications. In the study of Cogendez et al. (7), 151 patients underwent diagnostic hysteroscopy following a missed or an incomplete abortion, endometrial polyps were identified in 12 patients (7.9%). Also, Elsokkary et al. (8) examined 200 women with a history of three or more consecutive unexplained miscarriages before 20 weeks. He found that 18 patients suffered from endometrial polyps. Our findings coincide with those reports in demonstrating that endometrial polyps can be identified during the post-abortion period.

The role of endometrial polyps in the etiology of subfertility and early pregnancy loss among premenopausal women has been extensively debated in the literature (10–14). Endometrial polyps, as well as other structural pathology in the uterine cavity, may lead to subfertility or implantation failure (9). Endometrial polyp may occur as single or multiple lesions (15), however, we documented an even low rate of multiple lesions. This might be attributed to limited normal uterine environment, affecting embryo implantation. Additionally, our data revealed that no case of endometrial polyp exceeded 2 centimeters in size. Consistent with existing literature (16, 17), endometrial polyps of smaller size (<2 cm) seem not to decrease pregnancy rates but they may increase risk of spontaneous abortion. We suspect that smaller polyps may be more prevalent or larger polyps are more easily detected during routine assessments.

Interestingly, we documented a high rate (69.2%) of nulliparous individuals in study group and lower gravidity in the endometrial polyps group compared to controls. This can likely be explained by the fact that women with multiple gravidities have ever undergo ultrasound scans to inspect uterus, while nulliparous women are less likely to undergo ultrasound scans to inspect the uterus prior to pregnancy. Moreover, it is plausible to speculate that endometrial polyps might be present prior to pregnancy but undetected. Although sonographic evaluations for endometrial polyps were uniformly unremarkable in all women during pregnancy in our study. The possibility of endometrial polyps presenting prior to conception can't be excluded completely. However, reports of endometrial polyps identified on ultrasound scans during pregnancy are rare. In the study of Memtsa et al. (18), the ultrasound findings of endometrial polyps diagnosed in the first trimester of pregnancy were described. The detection of endometrial polyps during pregnancy is challenging.

Incomplete abortion traditionally been defined as RPOC failing to evacuate the uterus completely. Incomplete abortion may be managed expectantly or treated surgically or medically. Expectant management is an option for stable women with sub-menstrual bleeding who prefer to wait for the RPOC to exit the uterus naturally in the absence of medical intervention (19). However, expectant management is limited as a treatment option, as risks such as uterine bleeding, infection or failure to conceive thereafter, have been shown to outweigh the benefits (20–22). Transvaginal sonography (TVS) is considered a simple examination with good accuracy for most uterine cavity abnormalities (23), with 83.3% of sensitivity in detecting endometrial polyp (24) and 94% sensitivity in detecting RPOC (25). The sonographic diagnosis of RPOC is based on the appearance of hyperechoic material in the endometrial cavity (26). However, no case of endometrial polyps was identified before subsequent surgical intervention in post-abortion in our study. The identification of endometrial polyps following abortion presents a complex diagnostic scenario.

Previous studies (7, 8) exclusively included patients who suffered recurrent pregnancy loss for evaluation. In contrast, our study innovatively encompassed all types of abortion, with particular emphasis on differentiating endometrial polyps from RPOC in incomplete abortion cases. Our study revealed that endometrial polyps following abortion presents with lower blood flow, smaller size, lower level of serum β-hCG and longer time interval to subsequent surgical intervention compared with RPOC. This suggests that endometrial polyps are frequently misdiagnosed as incomplete abortions, which are typically managed expectantly or medically. For women with lower gravidity (particularly nullipara) presenting with persistent intrauterine hyperechogenicity and sub-menstrual bleeding after abortion, endometrial polyps should be included in the differential diagnosis, especially in cases managed expectantly or medically where the clinical presentation may mimic incomplete abortion. Surgical intervention should be arranged to minimize diagnostic delays and unnecessary interventions.

Incomplete removal of the residua is more likely to occur during repeated conventional curettage (27, 28). Post-abortion hysteroscopy is a simple and efficient tool for the early diagnosis and treatment of congenital and acquired intrauterine pathology after abortions (29). Hysteroscopy has the advantage over curettage in that it allows the direct visualization of the uterine cavity. Such direct visualization has several potential advantages, including localization of pathology, determination of the adequacy of dilatation and curettage and of biopsy, and precise diagnosis of uterine anomalies. In addition, hysteroscopic polypectomy is a safe procedure with low complication rate. Menstrual pattern was normalized in the majority of patients after hysteroscopic polypectomy.

### Limitations

As in any clinical study, this study has its limitation. A major limitation is the insufficient sample size and retrospective design from a single-center study. However, we believe a large multicenter study can easily be conducted to increase the sample size and validate the findings in the future.

## Conclusions

In summary, the clinical findings of the present study show that endometrial polyps should be considered as a potential cause of uterine abnormalities after abortion. In stable women after abortion referring to expectant or medical management, intrauterine occupancy present with lower likelihood of abundant blood flow on doppler, low serum β-hCG levels and prolonged retention after abortion, particularly in those with lower gravidity, suspicion for endometrial polyps should be raised rather than for incomplete abortion. Hysteroscopic evaluation is recommended to confirm the diagnosis and guide appropriate intervention, avoiding unnecessary management for presumed retained products of conception.

## Data Availability

The original contributions presented in the study are included in the article/Supplementary Material, further inquiries can be directed to the corresponding authors.
